# Novel approach to enhance the efficiency of mortar against radiations by co-incorporation of lead and boron compounds with calcium chloride

**DOI:** 10.1038/s41598-024-85019-2

**Published:** 2025-01-06

**Authors:** Hussein Al-kroom, Ahmed S. Ouda, Wageeh Ramadan, Mahmoud Gharieb, Mudar Hamsho, Hamdy A. Abdel-Gawwad

**Affiliations:** 1 Department of Civil Engineering, School of Engineering, The University of Jordan, Amman, 11942 Jordan; 2 Raw Building Materials Technology and Processing Research Institute, Housing & Building National Research Center, HBRC, Cairo, Egypt; 3 Radiation Protection and Safety Department, Hot Labs. Centre, Egyptian Atomic Energy Authority (EAEA), P.O. Box 13759, Cairo, Egypt; 4 Department of Civil and Environmental Engineering, Qatar University, Doha, Qatar; 5 Department of Building Materials and Construction Chemistry, Institute of Civil Engineering, Technische Universität Berlin, Berlin, Germany

**Keywords:** Heavy-density mortar, Chemical additives, Leachability, Radiation parameters, Chemistry, Engineering, Materials science, Physics, Nuclear physics

## Abstract

Fabrication of heavy density mortar using aggregates reinforced with available solid inorganic chemical additives is of a great importance as a protective layer to mitigate radiations in nuclear facilities. The effect of lead oxide and borax decahydrate on the hydration kinetics was evaluated by determining setting time, leachability and compressive strength. To speed up the reaction, 0.5% calcium chloride was added to all formulations, and then the results were compared to their blank counterparts. Once, the optimal compositions were explored, another batch of mortar were designed to increase efficiency against radioactive sources with different photon energies. After 28 days, bulk density, linear attenuation coefficient, half-value layer, tenth-value layer, and mean-free path in the field of ^137^Cs and^51^Co were considered. Similarly, macroscopic effective removal cross-section was evaluated using radioactive source-^239^Pu-α-^9^Be. According to the previous literature, adding 0.2% PbO to cement is the optimal ratio without affecting hydration kinetics and phase composition. The study explored that, co-incorporation of PbO with 0.5% CaCl_2_ increases the ratio to 2.5%, while enhancing physico-mechanical and radiological characteristics against radioactive sources. Also, formulation incorporating 0.5% borax with 0.5% CaCl_2_ had superior attenuation against neutrons compared to other competitors.

## Introduction

Energy is one of the basic elements that affects the growth of any country’s economy in the modern era, as traditional energy sources, such as those based on coal, solar energy, and wind energy, are no longer sufficient to achieve global economic growth due to industrial progress and population growth. As a result, many countries are turning to establishing nuclear power plants to meet energy demand. In order to construct nuclear power plants, concrete structures must be made of materials capable of blocking all nuclear radiation^1–3^.

Concrete that functions as a barrier to protect against nuclear radiation must be made with specific raw materials in order to create special concrete structures that deal with radioactive sources or radioactive waste, as this concrete must have good shielding performance, high density, and high durability^4–10^. According to the energy and form of the emitted waves, nuclear facilities such as nuclear power plants, nuclear research centers, and radiation treatment centers can be categorized as emitting X-rays, alpha, beta, gamma rays, or fast and thermal neutrons^11–13^.

It is the shielding materials’ density that determines how much gamma rays attenuate. Preventing ray penetration is one of the most important aspects of designing a protective shielding system. The density of the shield material is the most important characteristic in stopping this penetration. Lead (Pb), tungsten (W), and iron (Fe) are examples of high-atomic-number materials and high-density metals that can shield high γ-ray energy, while aluminum plates can block low-energy forms of energy such as x, α, and β rays^14–16^. Lead is now a standard component in radiation protection system design because it has long been known to be a very effective material at shielding users from a variety of radiation sources. Due to its high atomic number, density, stability, ease of fabrication, high degree of application flexibility, and availability, lead is a great shielding material.

Hekal, et al.^17^ and Reddy and Reddy^18^ looked into the effects of lead (Pb) and lead nitrate, Pb(NO_3_)_2_, on the durability and mechanical properties of cement pastes. Lead addition to the cement pastes was found to cause a delay in the cement hydration (first and final setting times). The findings showed that at a lead (Pb) concentration of 2000 mg/L, the compressive strength somewhat increased, but that it decreased as the lead concentration increased. The results also demonstrate that the different cement pastes had a high degree of lead ion stabilization in the leaching that was obtained^19,20^.

Barbir, et al.^21^ looked into the effects of adding lead (II) oxide to ordinary Portland cement on its specific conductivity and hydration heat. The results showed that adding a higher concentration of lead (II) oxide caused the hydration processes to slow down and the heat values to drop. The addition of lead (II) oxide considerably postponed the appearance of maximum conductivity. It was established that w = 0.25 weight% of lead oxide (PbO) in the cement system is acceptable.

The gamma-ray shielding properties of the PbO-Al_2_O_3_-B_2_O_3_ and PbO-Al_2_O_3_-SiO_2_ glass systems were evaluated by Kaur and Singh^22^ was using the mass attenuation-coefficient, half-value layer, mean free path, and effective atomic number parameters. It has been inferred that the PbO addition improved the gamma-ray shielding properties.

Rapid neutrons are difficult to attenuate and have a high penetration rate. Good neutron shielding can be achieved by increasing the water content with specific materials or by utilizing additives with a high cross-section for neutron moderation or absorption. Due to their appropriate scattering cross-sections, hydrogen, iron, and carbon could be employed as moderators of fast neutrons. Li, et al.^23^ and Davraz^24^ examined the effects of boron compounds as cementitious composites on the various cement types’ hydration processes, as well as the controllability of these effects. The findings demonstrated that adding borax could successfully extend the setting time, and that as borax dosages increase, the retarding effect becomes more pronounced. Three effects of the borax incorporation are seen by SEM-EDS analysis: the hydration reaction rate is slowed down, gels are produced, and the slag’s surface is covered.

It is well known that boron, and in particular^10^B, has a very high thermal neutron absorption capacity. Because boron has a high cross section for capturing neutrons, it can be used in concrete to capture neutrons without generating highly energetic secondary gamma rays. And it was discovered that when the amount of boron in concrete increased, the mass attenuation coefficients increased linearly^25–27^. Yorgun, et al.^28^ demonstrated that as the doping rate of borax increases from 0 to 40% moll, the mass attenuation coefficient and effective removal cross section (ΣR) of fast neutron increase. Furthermore, it was noted that as the borax ratio increases, half value layer (HVL) values decrease. As would be expected, the presence of elements like H and B in the borax mineral content greatly raised the glasses’ ΣR values. Since boron carbide (B_4_C) is a good neutron absorber for use in nuclear reactors, it was added to concrete^29^. We looked into how the addition of B_4_C affected the concrete samples’ strength and physical characteristics. Also, they discovered that when B4C increased by up to 20% of the total weight of cement, the compression strength of concrete increased as well. Consequently, by lowering the concrete’s thickness, concrete containing B_4_C additives can take the place of traditional concrete for shielding nuclear reactors. The outcomes showed that the mechanical and physical qualities of concrete were enhanced by the addition of B_4_C.

After careful literature review, no research study was found that addressed the co-incorporation effect of inorganic solid admixtures on the physico-mechanical properties of cement pastes as well as strengthen the shielding performance of cementitious mortar against nuclear radiations emitted by various radioactive sources for use as protective layers in nuclear or related facilities. Accordingly, the primary goal of the present investigation is to enhance the shielding efficiency of heavy density mortar containing different percentages of solid inorganic chemical additives, including lead oxide and borax decahydrate against both gamma-rays and fast neutron, respectively. This was achieved by incorporating 0.5% calcium chloride into the prepared formulations. Initially, a batch of cement pastes was designed by adding 0.5, 1, 1.5, 2 and 2.5% of PbO, and 0.5, 1, 1.5 and 2% Na_2_B_4_O_7_.10H_2_O to OPC. The setting times and heavy metal leaching susceptibility were determined after 28 days of curing, while the compressive strength was studied for 90 days. After achieving the optimum proportions of chemical additives in the first section, a batch of cement mortar with a ratio of 1:3 OPC to sand was manufactured. The bulk density and radiation parameters, including attenuation coefficients, half–value layer, tenth–value layer and mean free path, as well as fast neutron, were studied with the radioactive sources Cesium-^137^Cs (662 KeV), Cobalt-^51^Co (1173 and 1332 KeV) and ^239^Pu-α-^9^Be. In this work, the researchers tried to provide an innovative solution to the problem of delayed hardening time and retarding effect of chemical additives on cement hydration, with increasing the efficiency of cementitious mortars against nuclear radiations, while introducing new information in this regard.

## Experimental aspects

### Materials

**Fig. 1:**
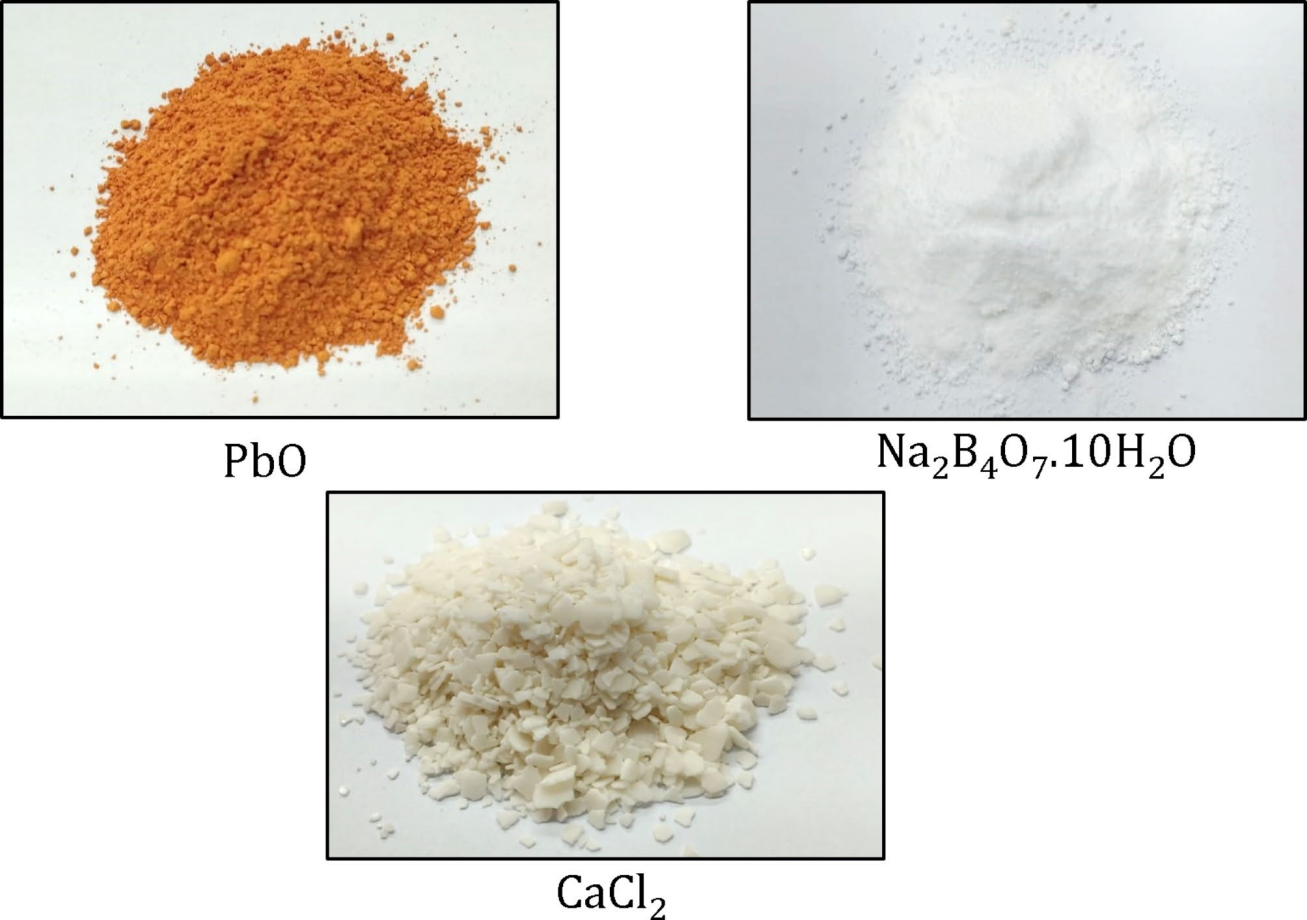
Images of inorganic solid chemicals.

According to the standard specification BS EN 197 − 1^30^, ordinary Portland cement (CEM I 42.5 N) with specific gravity of 3.15, supplied by Suez Cement Company, Egypt, was nominated as a binder. Table 1 outlines the chemical, physical and mechanical compositions of OPC. In some mixtures, different proportions of lead oxide (LO), borax decahydrate (BD) and calcium chloride (CC) with a purity of 99% were added to increase the shielding efficiency against gamma rays and fast neutrons. These admixtures were provided by Sigma Aldrich Company. The oxide content of LO is 98% PbO, 2% loss on ignition and density of 9.5 g/cm^3^. Its particle size is 1.88 μm. While, the oxide content of BD is 36.2% B_2_O_3_, 16.6 Na_2_O, 47.4% loss on ignition and the density is 1.73 g/cm^3^. Its particle size is 168 μm. Figure 1 illustrates images of the solid chemical additives.

**Table 1:** Chemical, physical and mechanical compositions of OPC (wt. %).

SiO_2_	Al_2_O_3_	Fe_2_O_3_	CaO	MgO	SO_3_^−−^	Na_2_O	K_2_O	L.O.I.	Total
21.26	4.49	3.49	63.81	2.02	3.11	0.14	0.09	1.57	99.98
Blaine fineness, cm^2^/g			3375						
Setting times, mins			Initial: 155						
			Final: 240						
Compressive strength, MPa			7 days: 231						
			28 days: 347						

### Preparation and mix design

To investigate the effect of both PbO and Na_2_B_4_O_7_.10H_2_O on the performance of cement pastes and mortars, and according to the mix composition of mixtures outlined in 2 & 3, respectively, two principal classes of formulations were fabricated. The control sample was comprised 100% OPC (CC0). The first category includes five mixtures that were prepared by the addition of 0.5%, 1%, 1.5%, 2% and 2.5% lead oxide to OPC. The codes (LO_0.5_, LO_1_, LO_1.5_, LO_2_ and LO_2.5_) were assigned to the indicated mixtures, respectively. While the second category includes four mixtures that were synthesized by the addition of 0.5, 1, 1.5 and 2% borax-decahydrate to OPC. Their codes are BD_0.5_, BD_1_, BD_1.5_ and BD_2_, consecutively. To enhance the mechanical strength of mixtures and to overcome the elongation of setting time caused by chemical additives to OPC, further batches of samples having the same composition were prepared with the addition of 0.5% CaCl_2_ (CC), by weight to the binding precursor. Also, five mortar mixtures were prepared, which were coded as M_0_, M_1.5LO_, M_1.5LO0.5CC_, M_0.5BD_ and M_0.5BD0.5CC_ (Table 2). In accordance with the requirements of specification ASTM C305^31^, the primary constituents were mixed in a saturated surface dry conditions, then the mixing water was gently poured into the dry powder in a 5 L mixing bowl from 3 to 5 min. After the mixture becomes homogeneous, the fresh paste was casted into cubic steel moulds with dimensions of 25 × 25 × 25 mm similar to^32^. To avoid airflow and dust, the cubes were covered with plastic sheets. On the day following casting, the cubes were removed and immersed in tap water at different ages until testing times.

**Table 2:** Mix composition of cement mortar incorporating solid inorganic additives (wt. %).

Mix ID	OPC	Sand	LO	BD	CC	w/b ratio
M_0_	1	3	–	–	–	0.56
M_1.5LO_	1	3	1.5	–	–	0.56
M_1.5LO0.5CC_	1	3	1.5	–	0.5	0.56
M_0.5BD_	1	3	-	0.5	–	0.56
M_0.5BD0.5CC_	1	3	-	0.5	0.5	0.56

### Testing approaches

**Fig. 2:**
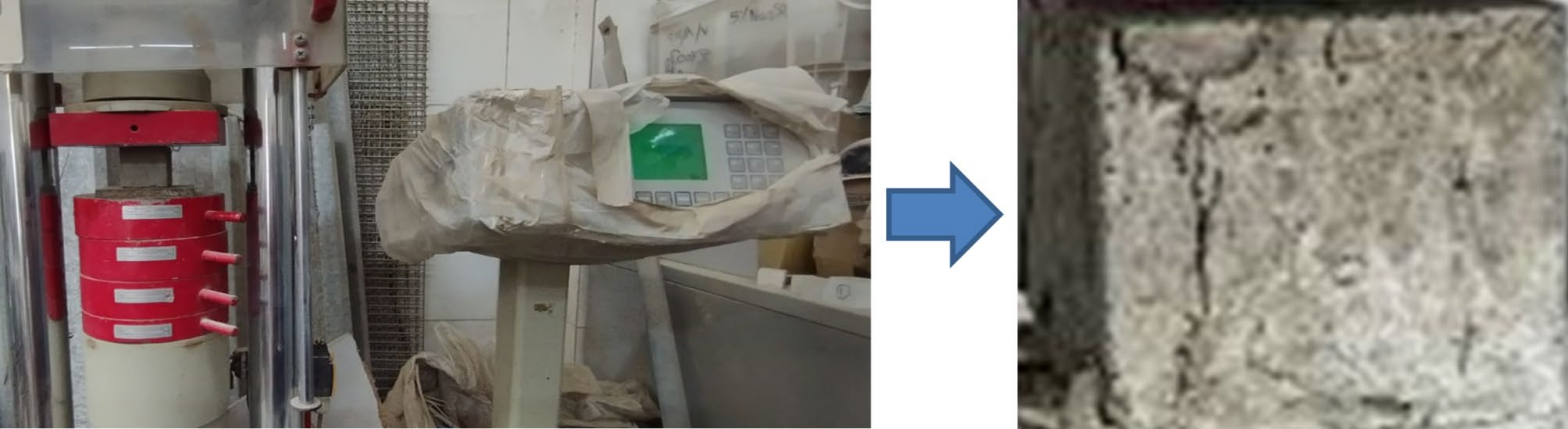
Photo of cube crushing machine during the compression testing.

After curing at ages of 7, 28, 56 and 90 days, three cubes of each hardened mixture were subjected to compressive strength procedures according to ASTM C109/C109M^33^. Figure 2 is an illustration of the compression test procedure. To avoid exposing the samples to additional moisture, the crushed pieces were soaked in a mixture of acetone and methanol (1:1, by volume), and then dried at 70 °C for 2 h. The next step was to keep samples in plastic bags and store them in a desiccator filled with silica gel for further investigations. After identifying the optimal mixtures, initial and final setting times of fresh pastes were determined using Vicat needle test method as per ASTM C191^34^.

Leaching susceptibility of lead ions in various hardening cement mixtures was determined by measuring the concentration of leached ions in accordance with SW-846 Test Method 1311: Toxicity Characteristic Leaching Procedure. To verify the efficiency of shielding against nuclear radiation emitted from various sources, a cement mortar containing proportions of chemical additives, with dimensions of 50 × 50 × 50 mm was designed. For all specimens, the cement-to-sand ratio was estimated at 1:3, by weight. After 28 days of curing, a batch of mortars was subjected to bulk density measurements using the formula^35^:

$$Bulk{\text{ }}Density~\, = \,\frac{{Saturated{\text{ }}weight}}{{Saturated{\text{ }}weight{\text{ }}{-}{\text{ }}Suspended{\text{ }}weight}}\left( {g/cm^{3} } \right)$$

Before starting radiation measurements, another batch of the same blends was pushed into the oven to be dried at 105 °C for 24 h as recommended by^36^.

A sodium iodide NaI (Tl) scintillation detector (Canberra, model: 802) was used and connected to a computerized analyzer utilizing Genie 2000 (version 3.1) software to determine the attenuation coefficient of the gamma rays for the specimens. Cesium-^137^Cs (662 KeV) and Cobalt-^51^Co (1173 and 1332 KeV) photon energies of 5µCi were employed. Because of its strong light emission and excellent compatibility with photomultiplier tube sensitivity, sodium iodide (NaI) was used in gamma-ray spectrometers to provide great, economical energy resolution. By comparing the specimen, fractional radiation intensity (I) at the appropriate thickness (x) with the initial intensity (I_o_), the linear attenuation coefficient (µ) of gamma rays was determined^37–39^. According to the Lambert-Beer, the intensity equation is described by Eq. (1);

1 $$I{\text{ }} = {\text{ }}I_{o} e^{{ - \mu x}}$$

The important gamma transmission parameters [half-value layer (HVL) and tenth-value layer (TVL)] are the absorber thicknesses (x) that may reduce the intensity of gamma rays to half and tenth their value, respectively. The mean free path (MFP) is defined as the mean of the gap between two subsequent photon interactions^40^. Equations (2), (3), and (4) are used to figure out such parameters as:

2 $$HVL{\text{ }} = {\text{ }}\frac{{\ln 2}}{\mu }$$

3 $$TVL{\text{ }} = {\text{ }}\frac{{\ln 10}}{\mu }$$

4 $$MFP{\text{ }} = {\text{ }}\frac{1}{\mu }$$

**Fig. 3:**
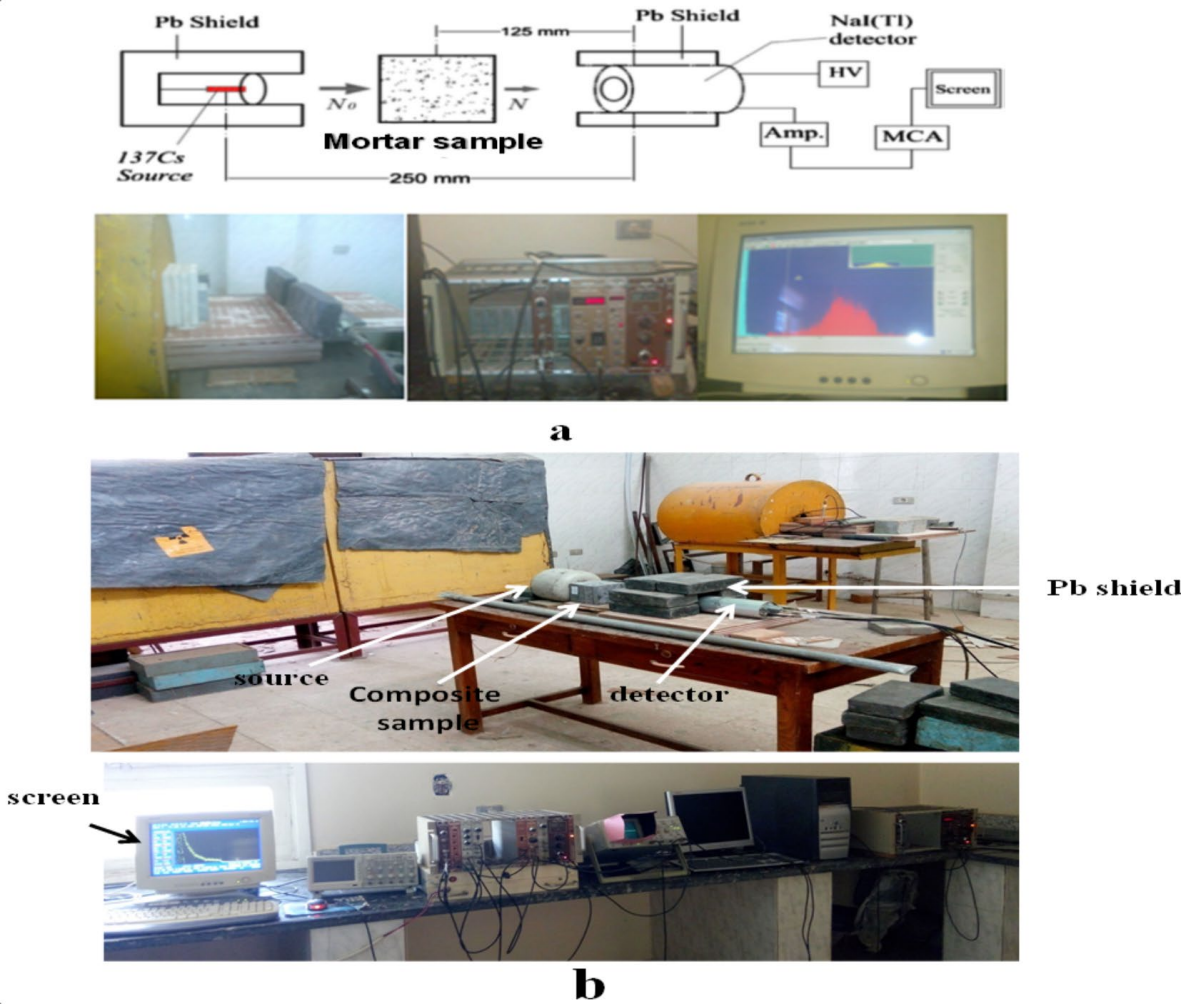
The schematic representation of the experiment: gamma-rays (**a**),fast neutrons (**b**).

Higher neutron fluence, a compressed design, and a reasonable photon contribution in the radiation scope are the characteristics of the 5 Ci radioactive source (^239^Pu-α-^9^Be). The stilbene scintillator (4 × 4 cm crystal), which was attached to a photomultiplier tube EMI-2232B for its high degree of time definition and high time resolution, monitored the fast neutrons sent spectra through the tested substances. The experimental measurements have been carried out to detect the spectrum of fast neutrons at Labs. For Developing Nuclear Techniques to Detect Landmines and Illicit Materials, Nuclear Research Center, Egyptian Atomic Energy Authority (EAEA). The source was positioned within a lead collimator that had a 10 mm aperture diameter to provide a narrow beam suitable for measuring procedures. To attain the proper count rate, the detector was positioned 300 mm from the radiation source, and samples with varying thicknesses (50–150 mm) were arranged in front of the detector. The recoil protons’ pulse amplitude distributions that transformed into the energy spectra of fast neutrons were measured using the algorithm NSPEC, which is based on double discrimination and matrix correction techniques^41–44^. The experimental setup for gamma rays and fast neutrons, respectively, is shown in Fig. 3a,b in a manner similar to that used by^40^.

The macroscopic effective removal cross-section (∑_R_) can be calculated using Eq. (5);

5 $$I\, = \,I_{0} e^{{ - \sum\nolimits_{R} X }}$$

In which, I_o_ and I are the initial and final intensities measured behind the investigated samples with different thicknesses (x), respectively.

## Results and discussion

### Effect of LO and BD additives on water-to-cement ratio

The results listed in Table 3 show the w/b values of cement pastes impregnated with LO and BD powders, i.e., batch No.1. With insight, an inverse proportion between these chemicals and w/b was observed; pointing out that the higher the percentage of the chemical in the matrix, the lower its water content, and vice versa. The w/b values of cement pastes containing LO compound ranged from 0.282 to 0.297; with a decrease of 10.47%-to-5.71% lower than its reference counterpart. On the opposite side, inclusion of BD at different ratios almost reduced the water-to-binder ratio from 0.298 to 0.293. The rate of decline compared to the control mix was 5.39–7.86%. Concerning the issue, Barbir, et al.^21^ acknowledged that the cement matrix containing a dosage higher than 0.25 wt% PbO helps slow down the hydration reaction, resulting in a lower amount of heat released; therefore, the reaction becomes less exothermic. In a similar manner, Ataie^45^ reported that borax reduces the dissociation degree of OPC molecules. By comparison, it was found that the pastes mixed with BD material showed higher water of consistency (i.e., higher w/b ratio) than their analogues mixed with chemical LO under the same casting conditions. For instance, the pastes that incorporated 0.5-2% BD provided water-to-cement ratio of 0.34–1.37%, respectively more than comparable mixtures with LO. With respect to the corresponding pastes containing 0.5% CC, i.e., batch No. 2, a decrease in the w/b ratio was also observed compared to the reference ones. The effect of incorporating CC with other additives in cement matrix has an obvious effect on the water consistency. As predicted, a remarkable increase in w/b ratio was spotted for all pastes doped with CC. The addition of 0.5% CC increased the w/b values of pastes mixed with LO powder approximately by 4.04%, 2.72%, 2.73%, 1.72% and 3.54%, respectively for mixtures (CC_0.5_LO_0.5_, CC_0.5_LO_1_, CC_0.5_LO_1.5_, CC_0.5_LO_2_, CC_0.5_LO_2.5_) compared to their CC-free counterparts. While, the inclusion of CC in pastes doped with BD powder increased the w/b ratio by 3.02%, 1.69%, 0.67% and 0.68%, consecutively for pastes (CC_0.5_BD_0.5_, CC_0.5_BD_1_, CC_0_._5_BD_1.5_, CC_0.5_BD_2_) versus the reference. Cheeseman and Asavapisit^46^ reported that the incorporation of calcium chloride reduces the inhibition caused by the addition of 10% lead waste, by weight to cement system. Moreover, it reduces the decomposition and subsequent adsorption of Pb(OH)_3_^−^ ions on CSH surface, formed by the hydration process. Meanwhile, Kishar, et al.^47^ indicated that the merging of 0.25–0.75 CaCl_2_ into a cementitious matrix increases the combined water ratio for all studied pastes compared to its free-CaCl_2_ counterpart. They also claimed that this trend is ascribed to the enhancement of OPC hydration accompanied by formation of more products. This is in line with what was obtained by Bortoluzzi, et al.^48^, they attributed the increase in water content of the mixtures to the fact that calcium chloride is a hygroscopic compound, which may absorb a lot of water during the manufacture of pastes.

**Table 3:** Mix composition of cement pastes incorporating solid inorganic additives (wt. %).

Batch #1					
Mix ID	OPC	LO	BD	CC	w/b ratio
CC_0_	100	–	–	–	0.315
LO_0.5_	100	0.5	–	–	0.297
LO_1_	100	1	–	–	0.294
LO_1.5_	100	1.5	–	–	0.292
LO_2_	100	2	–	–	0.291
LO_2.5_	100	2.5	–	–	0.282
BD_0.5_	100	–	0.5	–	0.298
BD_1_	100	–	1	–	0.296
BD_1.5_	100	–	1.5	–	0.296
BD_2_	100	–	2	–	0.293
Batch #2					
CC_0.5_	100	–	–	0.5	0.340
CC_0.5_LO_0.5_	100	0.5	–	0.5	0.309
CC_0.5_LO_1_	100	1	–	0.5	0.302
CC_0.5_LO_1.5_	100	1.5	–	0.5	0.300
CC_0.5_LO_2_	100	2	–	0.5	0.296
CC_0.5_LO_2.5_	100	2.5	–	0.5	0.292
CC_0.5_BD_0.5_	100	–	0.5	0.5	0.307
CC_0.5_BD_1_	100	–	1	0.5	0.301
CC_0_._5_**BD**_**1.5**_	100	–	1.5	0.5	0.298
CC_0.5_BD_2_	100	–	2	0.5	0.295

### Effect of LO and BD additives on setting course of OPC

**Fig. 4:**
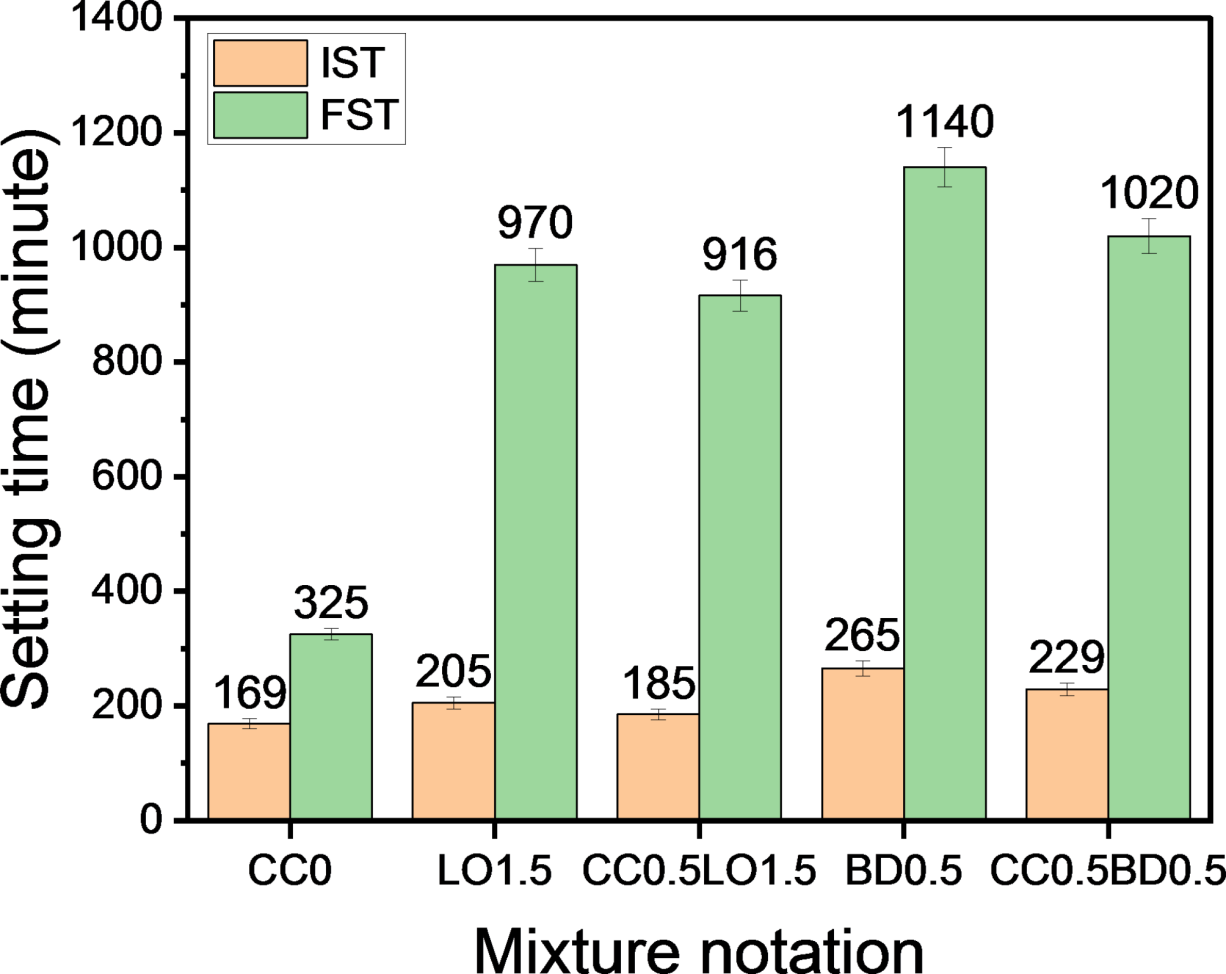
Effect of solid chemical additives on the setting times of fresh pastes.

Figure 4 shows the effect of chemical additives on OPC setting. It is evident that the initial and final setting times (IST & FST) of cement pastes are significantly delayed in the presence of different dosages of LO and BD. In the case of 1.5 wt% LO, IST and FST of pastes are 205 and 970 min, with an increase of 1.21 and 2.98 times, respectively longer than that of the reference mix. While its counterpart incorporating 0.5 wt% BD achieved an increase of 1.57 and 3.51 times, sequentially. Taking a closer look, adding BD in small doses, prolongs the setting times of OPC rather than those mixed with LO. This is in agreement with the trend reported by Keppert^49^, who studied the effect of lead nitrate (LN), lead sulfate (LS) and lead (II) oxide (LO) on the hydration kinetics of OPC pastes doped with 0.5%, 1%, 2% and 5%. He concluded that the LO causes longer IST and FST than other compounds. Likewise, Xu, et al.^50^ suggested that solid wastes loaded with lead compounds severely inhibited the hydration of OPC by forming complexes of lead(II) salts and silanol groups on top surface of clinker, which resulted in delaying the dissolution of C_3_S-based phases. Similarly, studies carried out by Niu, et al.^51^ indicated that trace proportions of lead(II) compounds added to OPC causes elongation of setting time. Concerning the effect of boron compounds on the hydration mechanism of cement, Tan, et al.^52^and Zajac, et al.^53^ revealed that the addition of borax to OPC retards the reaction and prolongs the setting time. According to study conducted by Hu, et al.^54^, the incorporation of borax into cement system can displace SO_4_ ^− 2^ ions in calcium sulfoaluminate to form a complex deposited on C_3_S surface, and thereby affecting on IST and FST as well as the mechanical properties. With respect to similar formulations mixed with 0.5% CC, a somewhat lower IST and FST values can be observed vs. non-containing ones. Accordingly, the mixture CC_0.5_LO_1.5_ revealed IST and FST setting courses lower than the assigned CC-free CC_0.5_ approximately 1.11 and 1.06 times, respectively. However, its competitor incorporating borax powder CC_0.5_BD_0.5_ showed a similar trend compared to its BD_0.5_ counterpart, as the IST and FST values decreased 1.16 and 1.12 times, consecutively. Undoubtedly, the addition of calcium chloride to cement mixtures loaded with LO and BD additives accelerated the hydration reaction, which helped reduce setting times; however, the indicated mixtures still achieved higher values than the reference samples without these additives. Based on the findings of Bortoluzzi, et al.^48^, the addition of 10% calcium chloride to white Portland cement slurries resulted in a reduction of IST by 50% and FST by 68.5% of the corresponding reference mixtures.

### Leachability of Pb from hardened mixtures

The results of the cement pastes incorporating different proportions of PbO were tabulated in Table 4. From the table, we can deduce the leachability of heavy metal lead in the OPC mixtures containing 2.5% of lead oxide. The results indicate that, the lead concentration is zero for all mixes except for LO_2.5_ mix that gives concentration of 0.0001 ppm. It is much less than the internationally permitted percentages, according to the SW-846 Test Method 1311: Toxicity Characteristic Leaching Procedure, the limit value for lead content is 5 mg.L^− 1^ and exceeding this value is considered waste as hazardous. This encourages the use of lead oxide with cement mixtures at a rate up to 2.5%. The cement mixtures work to entrapping and prevent the leakage of lead. The reason is come from the cement matrix and the closed micro-pore which to keep all cations of heavy metals^6,55^. The addition of CaCl_2_ affects the sealing of the cement matrix due to the increase of hydration and formation of C-S-H phase, so crucial for binding of heavy metals.

**Table 4:** Leaching susceptibility of pb from cement pastes (ppm).

Mix codes	Pb
CC_0_	0
LO_1.5_	0
LO_2.5_	0.00012
CC_0.5_LO_2.5_	0

### Impacts of LO and BD additives on compressive strength development

**Fig. 5:**
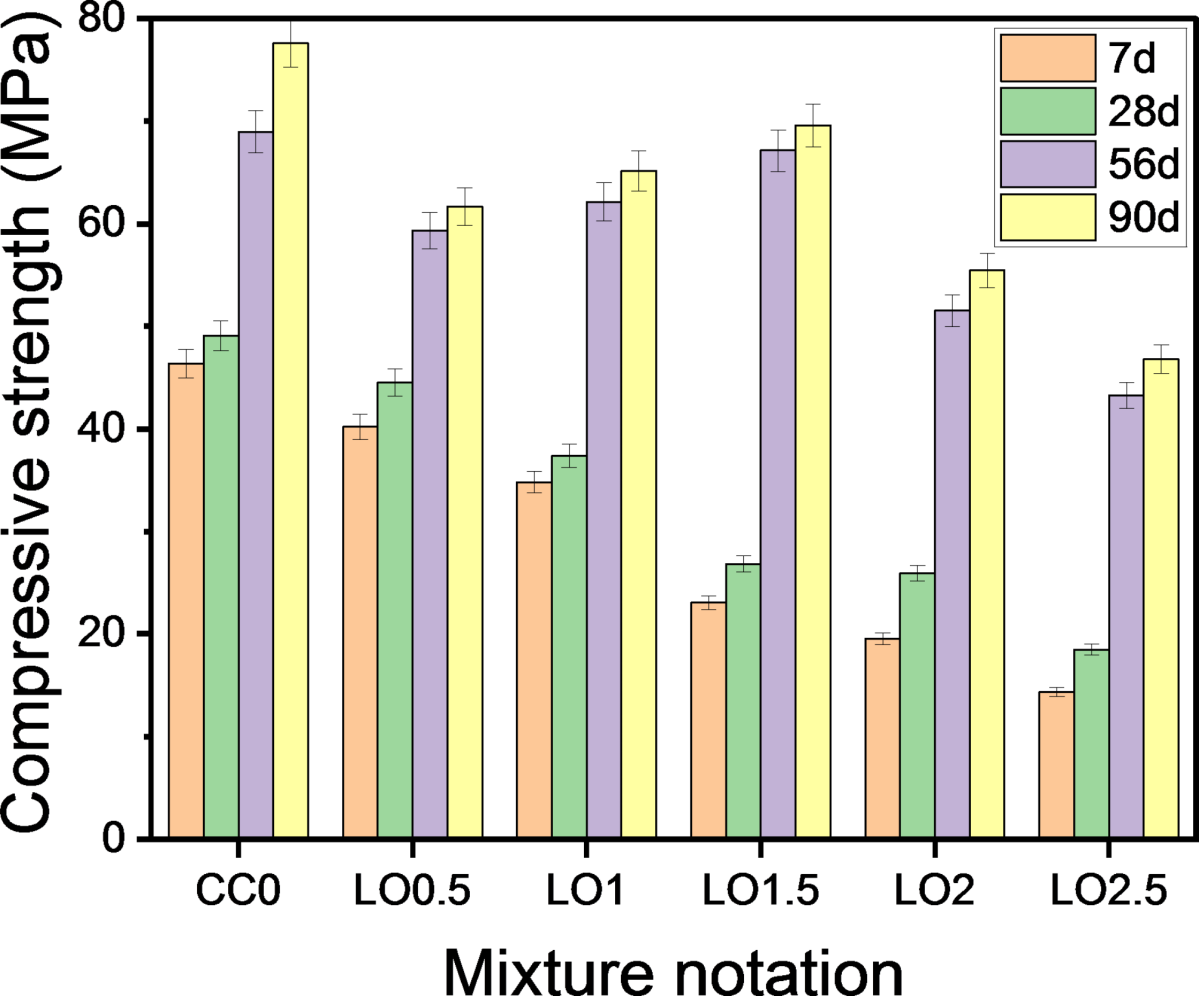
Compressive strength of specimens containing LO after curing times for 90 days.

**Fig. 6:**
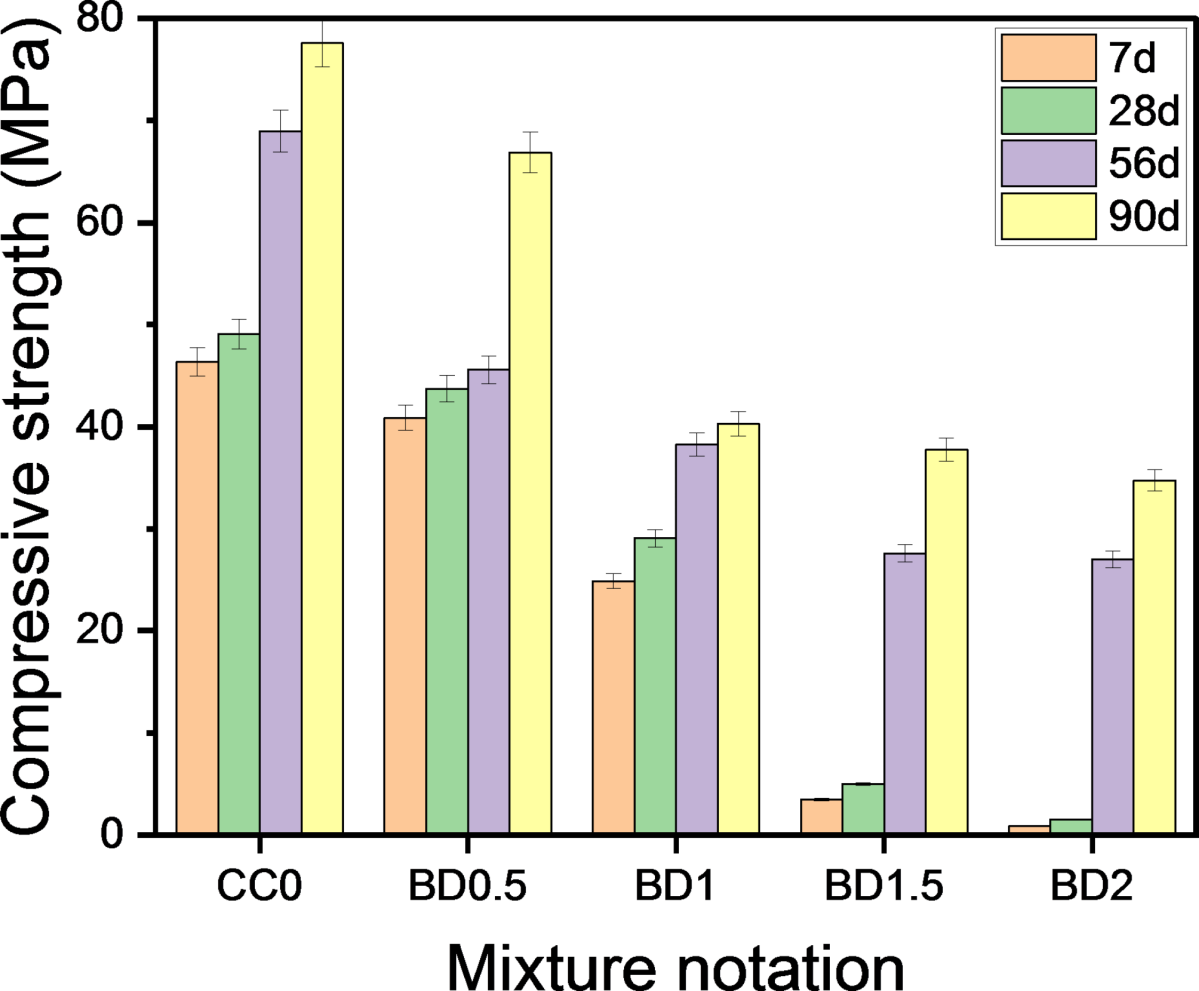
Compressive strength of specimens containing BD after curing times for 90 days.

To evaluate the effect of chemical additives on the mechanical performance of OPC pastes, the compressive strength of cement pastes containing different ratios of LO between 0.5 and 2.5% and their counterparts containing ratios between 0.5 and 2% BD were measured after curing ages 7, 28, 56 and 90 days compared to the control mixtures, and are represented in Figs. 5, 6, respectively. Remarkably, the strength of all investigated samples increases as time progresses; this trend is linked to the continuation of the hydration reaction and the formation of more products that resulted in a pore refinement. The incorporation of LO and BD additives into the cement matrix had a negative effect, as the compressive strength decreased at all curing ages with respect to the CC_0_ reference mixture. By comparing the different mixtures, it was found that there was a significant increase in the strength values of the paste admixtured with LO compared to that mixed with BD. Higher dosage of chemical additives resulted in further deterioration of the resistance values. The compressive strength exhibited apparently 13.31, 9.35, 13.96 and 20.52% reduction after 7, 28, 56 and 90 days, respectively when 0.5 wt% of LO was incorporated into the cement matrix vs. LO-free mixtures called CC_0_. Sequentially at similar immersion times, the addition of 1 wt% LO (LO1) indicated lower strength values of 24.93, 23.91, 9.90 and 16.01 corresponding to the normal OPC blend. When the added LO ratio exceeded 1.5 wt%, the pastes recorded a decrease in compressive strength of approx. 50.30, 45.37, 2.65 and 10.31%. Also, a reduction in compressive strength of about 57.85, 47.25, 25.30 and 28.55% was monitored for pastes incorporating 2 wt% LO at the respective ages. Likewise, a similar trend of results was explored with the inclusion of 2.5 wt., where the mixture LO_2.5_ exhibited a reduction of 69.01, 62.34, 37.29 and 39.69%. Based on what has been reported, Elnaggar, et al.^56^ concluded that the compressive strength of cement pastes decreases when the content of lead oxide and lead phosphate replacement in the blend increases from 0.1 to 0.5%, in particular at initial times against its reference counterpart. The exact opposite was observed at later times (i.e., 28 and 90 days), as the compressive strength increased when the ratio of 0.2% was incorporated. Asavapisit, et al.^57^ studied the properties of cement pastes mixed with 10%, 20% and 30% of lead and chromium hydroxides at a water/solid ratio of 0.45 by determining the FST, leachability and compressive strength at different curing ages. They reported that the strength of the hardened specimens decreases with increasing dose of heavy metals in the cement blend. From their point of view, the decline is ascribed to the adsorption of these compounds onto the outer surface of the clinker, resulting in the formation of less permeable layers that prevent the access of water to complete the hydration process. Through careful consideration of Fig. 6, it was found that infusing borax into cement pastes reduces the resistance values at all levels of addition, just like lead oxide, but with a sharp decline in values. Satisfactory strength values were observed only when 5% borax was included; however an additional dose of 1–2% resulted in a striking deterioration.

**Fig. 7:**
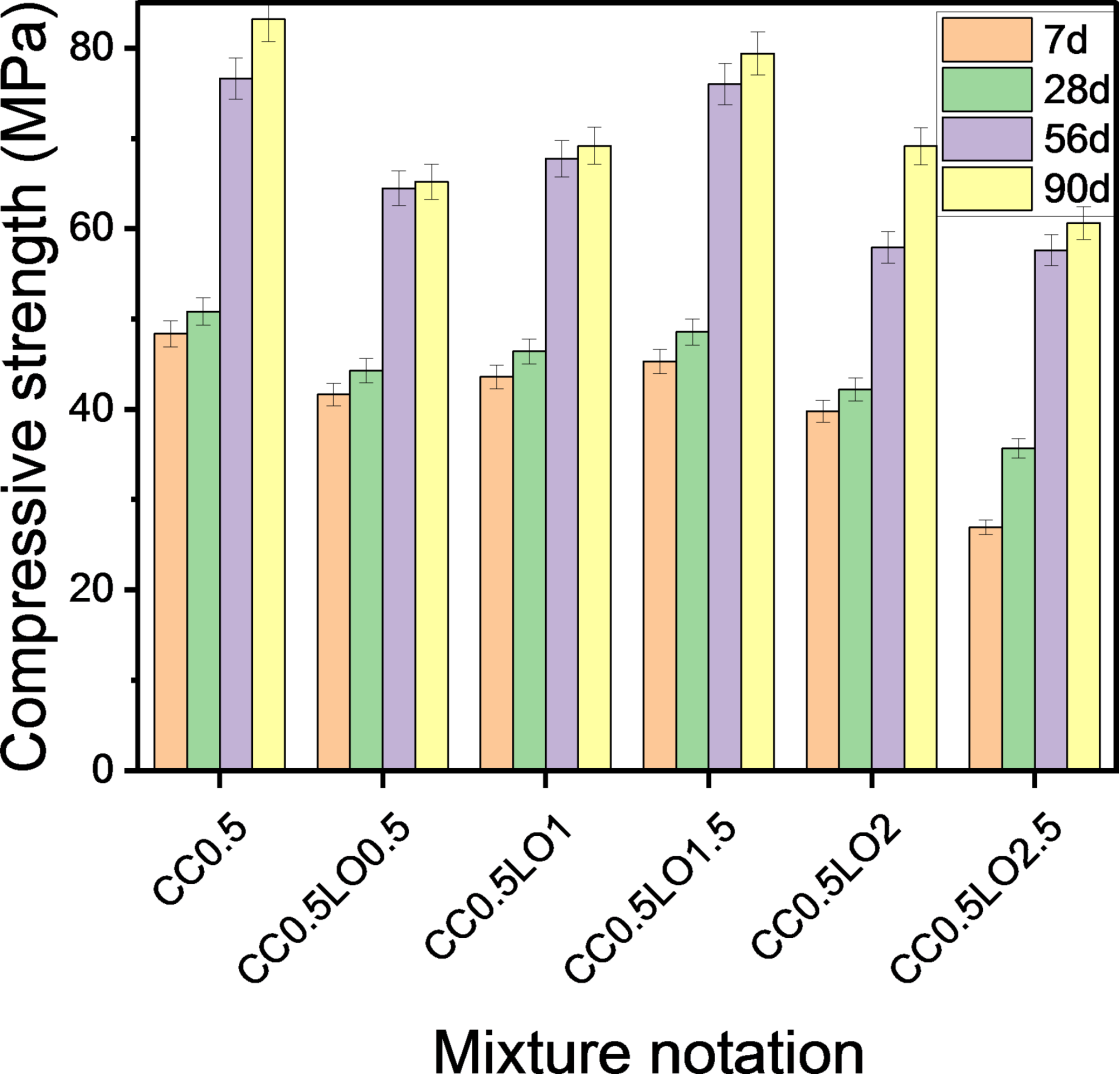
Effect of co-incorporation of LO and CC on compressive strength.

**Fig. 8:**
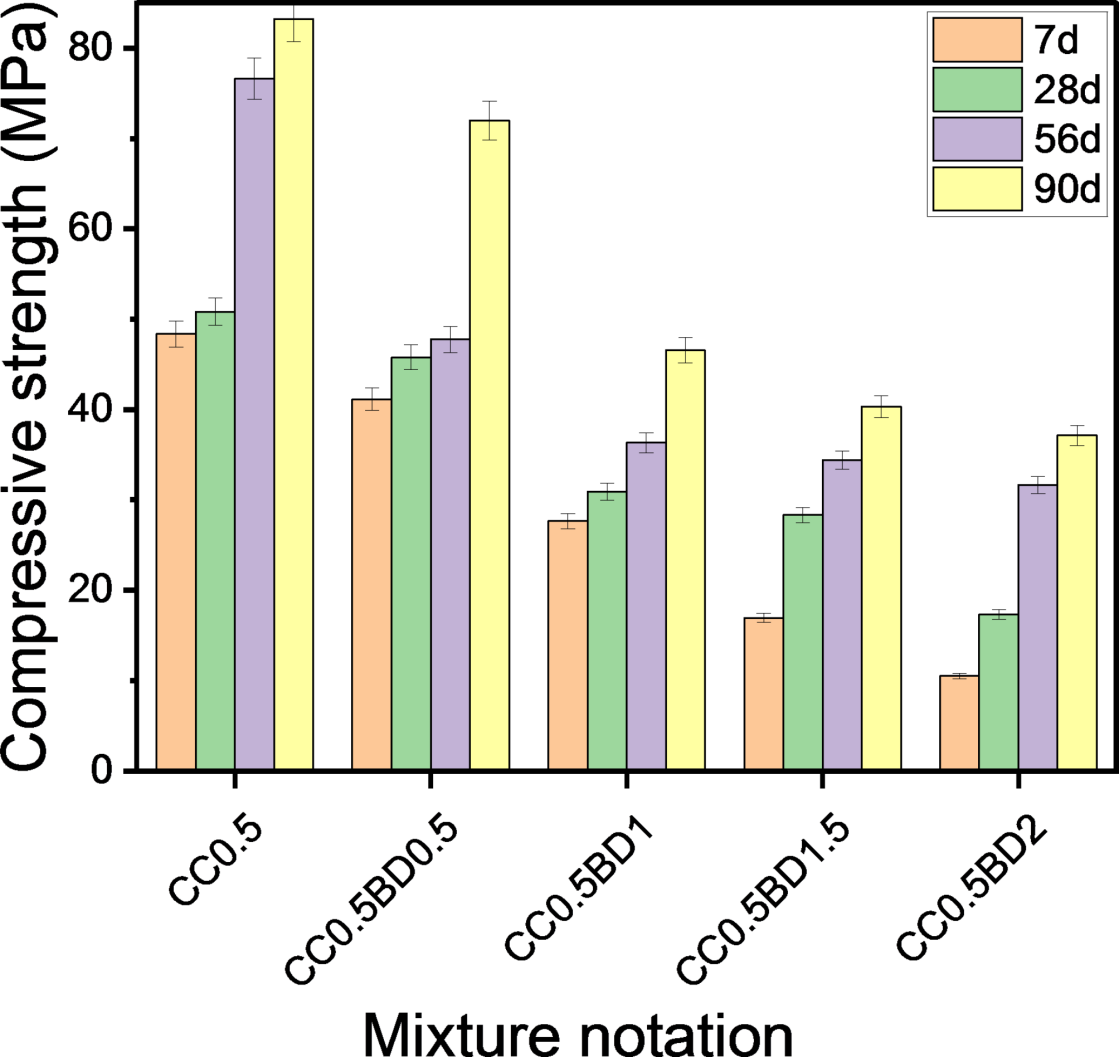
Effect of co-incorporation of BD and CC on compressive strength.

With the addition of CaCl_2_ to OPC pastes impregnated with chemical additives, an enhancement in compressive strength was detected (Figs. 7, 8). The results revealed that LO-cements with 0.5% CaCl_2_ provided higher strength values than pure cements at all curing periods. This is an evidence that the incorporation of calcium chloride accelerates the hydration reaction with strength development. According to Juenger, et al.^58^, CaCl_2_ accelerates the formation of calcium silicate hydrate (C-S-H), which acts as the primary binding agent. In the case of pastes termed as CC_0.5_LO_0.5_, CC_0.5_LO_1_, CC_0.5_LO_1.5_, CC_0.5_LO_2_ and CC_0.5_LO_2.5,_ an increase in compressive strength of 3.60%, 25.22%, 96.65% and 103.63%, respectively was achieved after 7 days of curing versus their pure pastes. It is worth noting that the highest strength reinforcement was recorded in subsequent ages, i.e. 90 days, as the concerned pastes provided an increase of 5.67%, 6.23%, 14.01%, 24.71 and 29.57%, consecutively. A similar trend of the results for pastes containing borax compound has been monitored. Although the merging of calcium chloride into these mixtures enhances their mechanical performance; however, they still record lower strength values than the reference ones (CC_0_, CC_0.5_). Through the foregoing, we can conclude that the accepted proportions of LO and BD that provide satisfactory compression resistance upon merging with CaCl_2_ are 1.5% and 0.5%, respectively.

### Phase composition

**Fig. 9:**
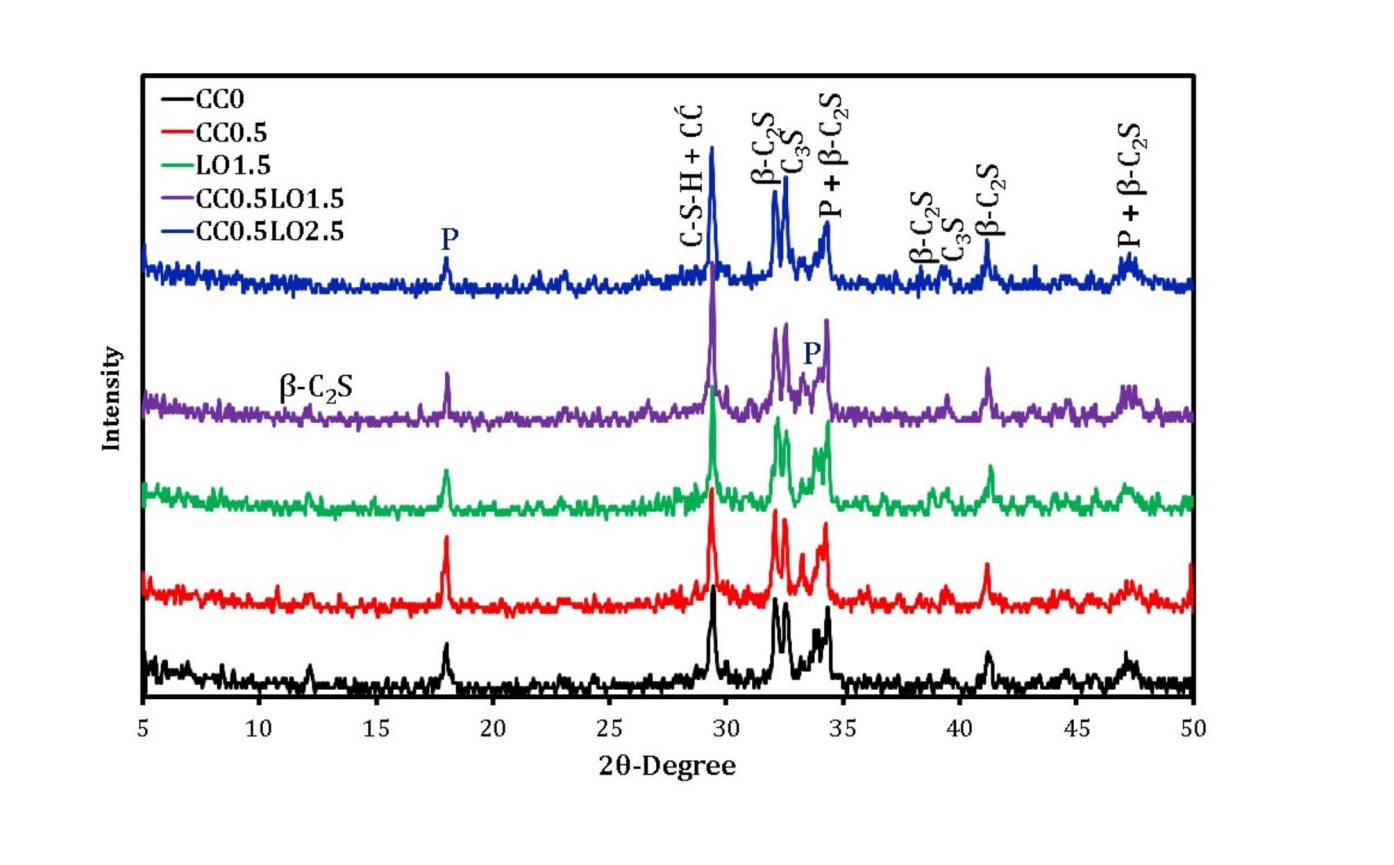
XRD spectrum of 28-hardened samples with co-incorporation of LO and CC.

Figure 9 represents XRD curve of 28-day hardened samples containing 1.5 and 2% LO with/without CC. For comparison, control mixtures CC0 and CC0.5 were included. The dominant peaks of portlandite (P) and crystalline calcium silicate hydrate (C-S-H) intercalated with calcite (CĆ) formed by hydration of cement grains, as well as non-hydrous compositions (C_3_S and β-C_2_S) were detected. With the inclusion of 1.5% LO, the intensity of both P and C-S-H phases is significantly reduced compared to their reference counterpart (CC0). Although the hydration reaction occurs, the characteristic peaks of C_3_S and β-C_2_S are still present. Surprisingly, their intensity increases with LO-loaded OPC pastes, suggesting that the process is severely delayed with lead incorporation and the retardation becomes much higher with increasing doses. A close relationship between the findings and previous literatures^51,59,60^. The researchers suggested that, the introduction of lead oxide into the cement matrix promotes the formation of Pb(OH)_2_ coating on the cement grains and thus limits their dissolution accompanied by retarding hydration and prolonging hardening times. In the case of mixtures containing 0.5%CC, the intensity of P increases, while that of the unreacted phases decreases, indicating that CaCl_2_ addition accelerates the hydration of C_3_S. This result is consistent with the findings reported by^47^. It is evident from the pattern that the crystalline peak of portlandite is decreased in intensity with the inclusion of 0.5% CC and 2.5% LO together in a single matrix compared to its other counterparts. This is also evidenced by the increased intensity of the peaks distinctive for the non-solubilized compositions. The incorporation of LO higher than the optimum dosage (1.5%) had no significant ability to accelerate hydration or the CC content was insufficient with the high levels of lead oxide.

**Fig. 10:**
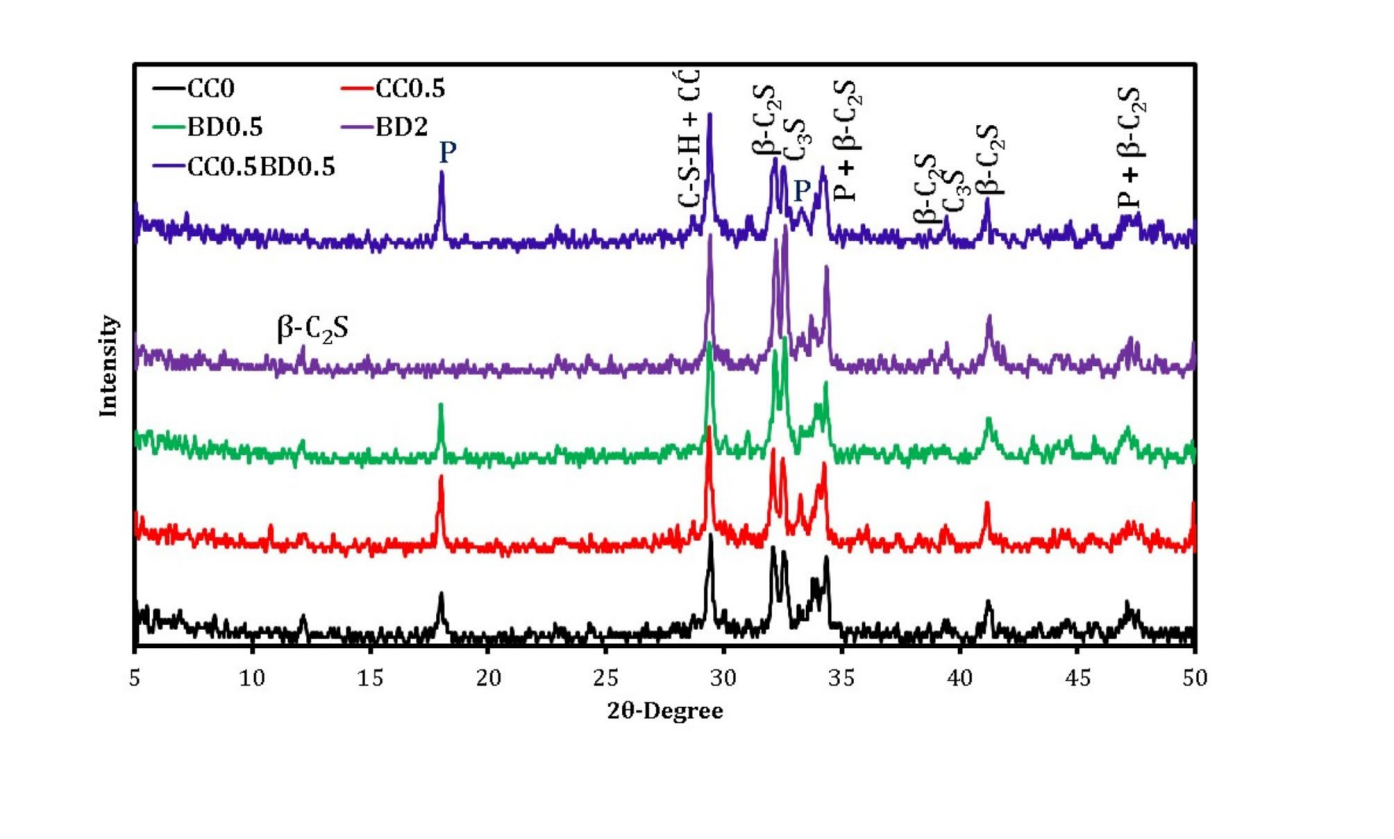
XRD spectrum of 28-hardened samples with co-incorporation of BD and CC.

The XRD analysis of pastes containing 0.5 and 2% BD after curing for 28 days was graphically investigated in comparison with the references CC0 and CC0.5 given in Fig. 10. In a similar manner, the spectrum indicates that, the principal hydration products consists of P and crystalline C-S-H phase in combination with CĆ as well as the non-hydrated phases. It is evident that, the characteristic peak of P disappears at 2θ of 17.81 and significantly diminishes at other positions with the addition of BD up to 2% (BD2). In contrast, the distinctive peaks of C_3_S and β-C_2_S are still present within the pattern, and their intensity also increases with the inclusion of higher doses of borax. This is conclusive evidence that the incorporation of large proportions of borax severely inhibits the cement hydration reaction and hence these mixtures have brittle physico-mechanical properties compared to their reference counterparts (CC0). Otherwise, the BD0.5 mixture exhibited relatively higher performance compared to its BD2 counterpart, as the hydration reaction was not significantly inhibited; this was proven by the increased intensity of the peaks characteristic for both portlandite or calcium silicate hydrate, with a decreased intensity of the peaks identified for the unreacted phases. For CC0.5BD0.5 blend, the co-incorporation of BD and CC has distinct effects on the hydration evolution. This is demonstrated by the increased intensity of portlandite peak and the consumption of more calcium silicate in additional C-S-H formation to enhance the structural integrity of cement matrix, accompanied by the improvement of its physico-mechanical properties.

**Fig. 11:**
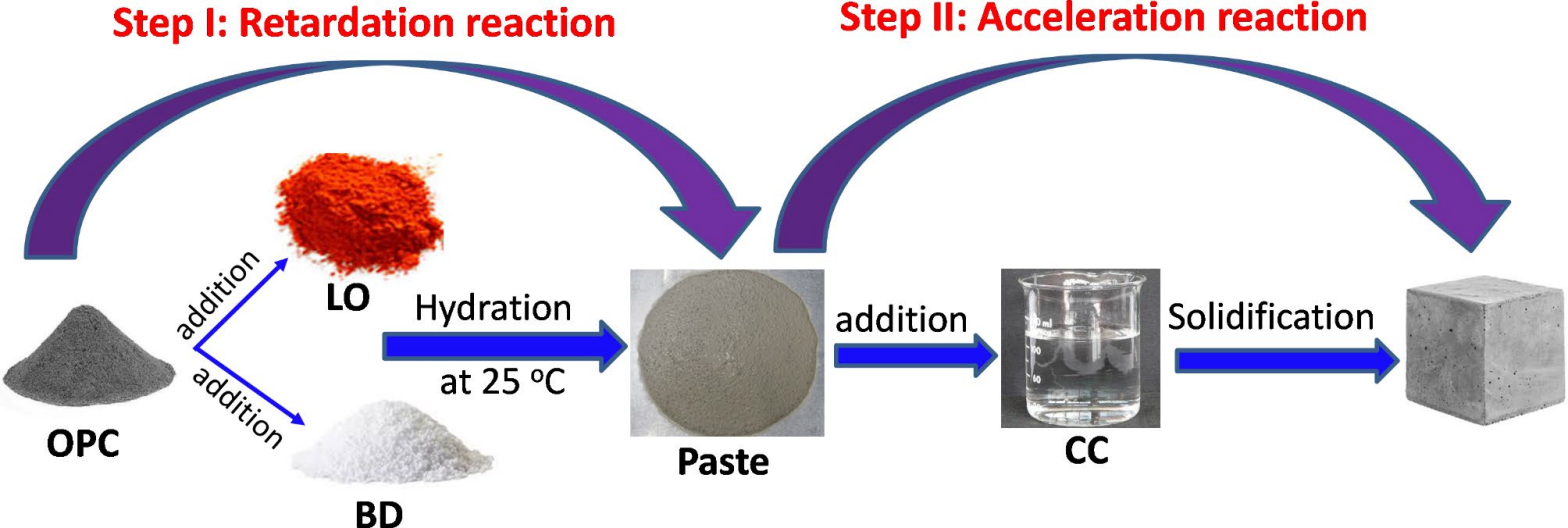
Schematic mechanism of cement pastes containing chemical admixtures in presence/absence of calcium chloride.

Similar to ElDeeb et al.^61^, Fig. 11 represents a schematic illustration of the reaction mechanism obtained upon mixing the chemical additives with cement in the presence or absence calcium chloride. For calcium chloride-free mixtures, the sole additives retard slow down the hydration reaction, and thus their physical and mechanical properties are affected compared to the reference counterparts. This stage is called “retardation reaction”. While, in the stage “acceleration reaction”, the co-incorporation of chemical additives with calcium chloride in a single matrix accelerates the hydration reaction of OPC, while enhancing the physico-mechanical characteristics.

### Effect of chemical additives on radiation mitigation

#### Attenuation of gamma-rays in the field of Cs and Co isotopes

**Fig. 12:**
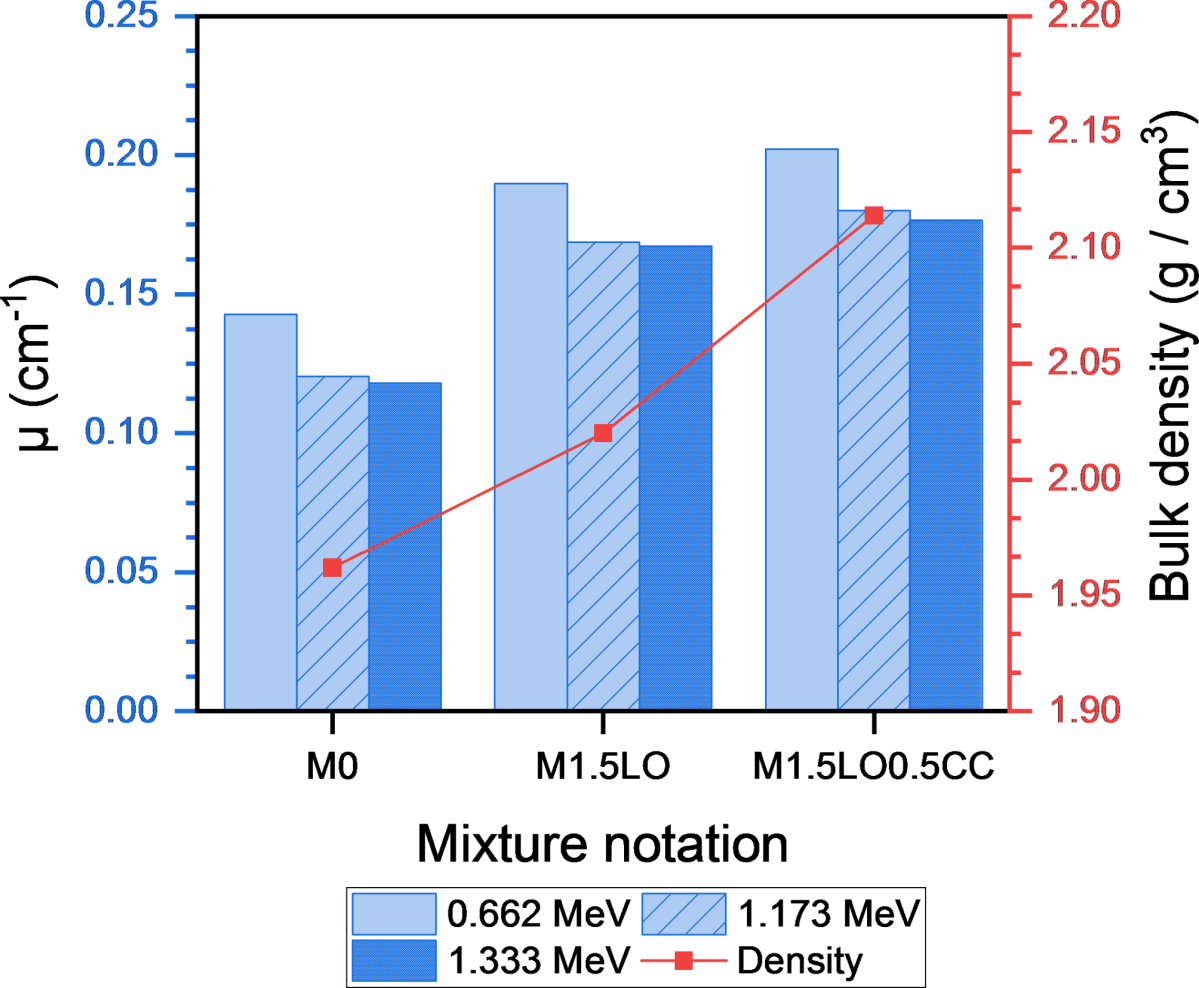
Effect of PbO and PbO/CC on the linear attenuation coefficient of cement mortar.

This section discusses the attenuation parameters, in terms of the determination of linear attenuation coefficient (µ, cm^− 1^), half-value (HVL), tenth-value layer (TVL) and mean-free path (MFP) of mixes M_0_, M_1.5LO_ and M_1.5LO0.5CC_ after exposure to isotopes of Cesium (^137^CS, photon energy: 0.662 MeV) and Cobalt^51^Co: photon energies: 1.173 MeV, and 1.333 MeV). The calculated practical data of µ are shown in Fig. 12. Regarding the issue, Ouda^62^ pointed out that, the protection coefficients, whether linear and/or mass-based depend largely on two variables: the hardened density of the mixture and type/source of gamma-rays. The findings revealed that, the linear attenuation coefficient increases with the inclusion of PbO in mortar matrix. As shown, the mix M_1.5LO_ exhibited µ value approximately 1.33 folds at photon energy 0.662 higher than the M_0_ mixture. The highest linear attenuation values were achieved with the co-incorporation of CC with LO in a single matrix, where the blend M_1.5LO0.5CC_ showed an increase of 1.41 folds in the field of gamma-ray irradiated by Cesium isotope compared to the reference counterpart. Figure 12 also describes the linear attenuation coefficient results for the same mixtures in the field of gamma-ray emitted by^51^Co (1.173 & 1.333 MeV). Closely, the µ values decrease with increasing the photon energy. Hence, the higher the photon energy, the lower the linear attenuation value^63^. The results of the cesium-irradiated samples were in agreement with their cobalt-irradiated counterparts, as the mixture M_1.5LO0.5CC_ showed the highest attenuation rate for gamma rays compared to the other mixtures. The increase at 1.173 was estimated to be 1.06 and 1.5 MeV higher than those recorded by M_1.5LO_ and M_0_, respectively. However, with increasing photon energy to 1.333 MeV, the optimized mixture M_1.5LO0.5CC_ recorded the highest µ values of 1.09 and 1.62 MeV. Implying that this mixture has higher hardened density (Fig. 12). This trend is attributed to the fact that the blend M_1.5LO0.5CC_ has a higher bulk density (ρ = 2.114) compared to the blend M_1.5LO_ (ρ = 2.020) and the control mix M_0_ (ρ = 1.962), respectively.

**Fig. 13:**
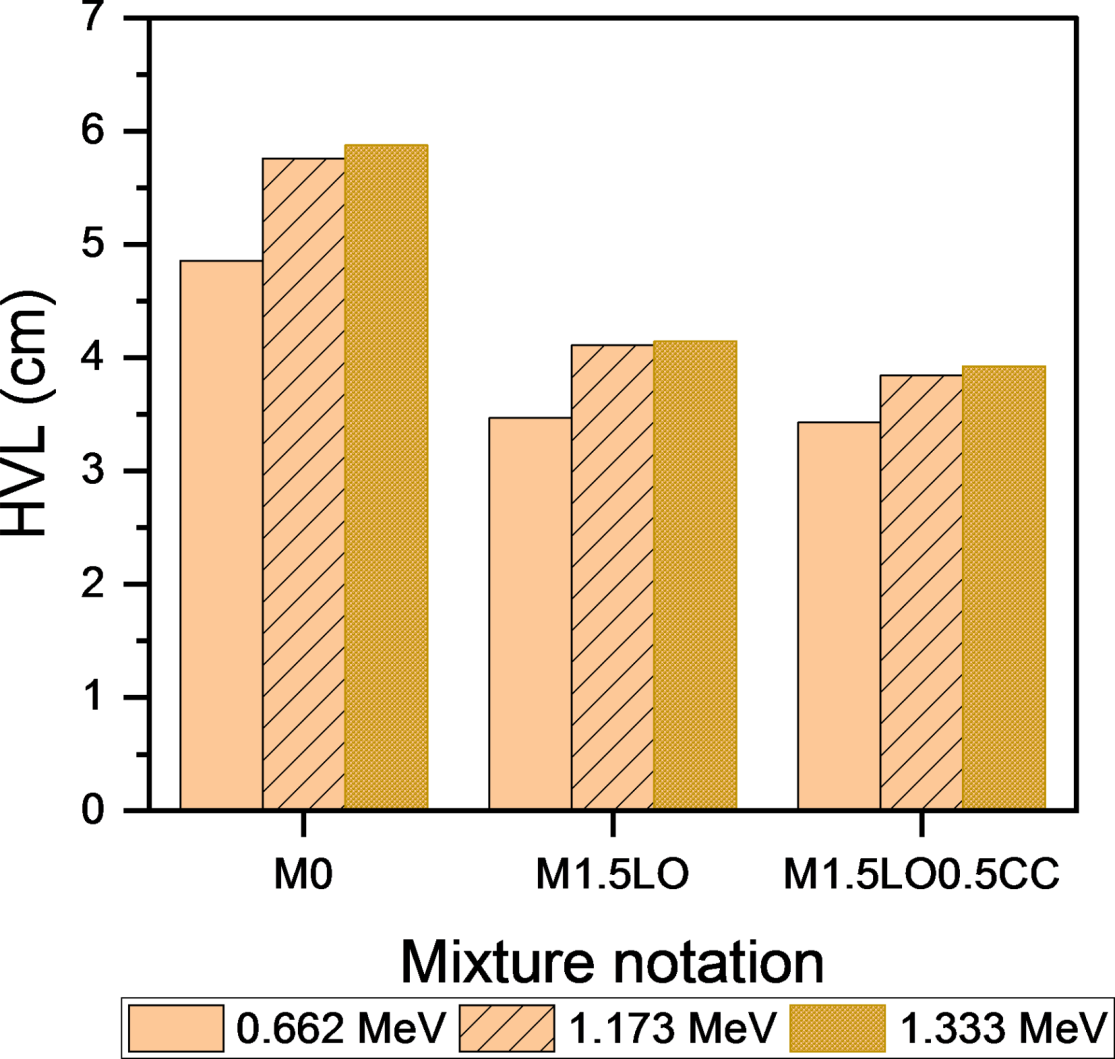
Effect of PbO and PbO/CC on half-value layer of cement mortar.

**Fig. 14:**
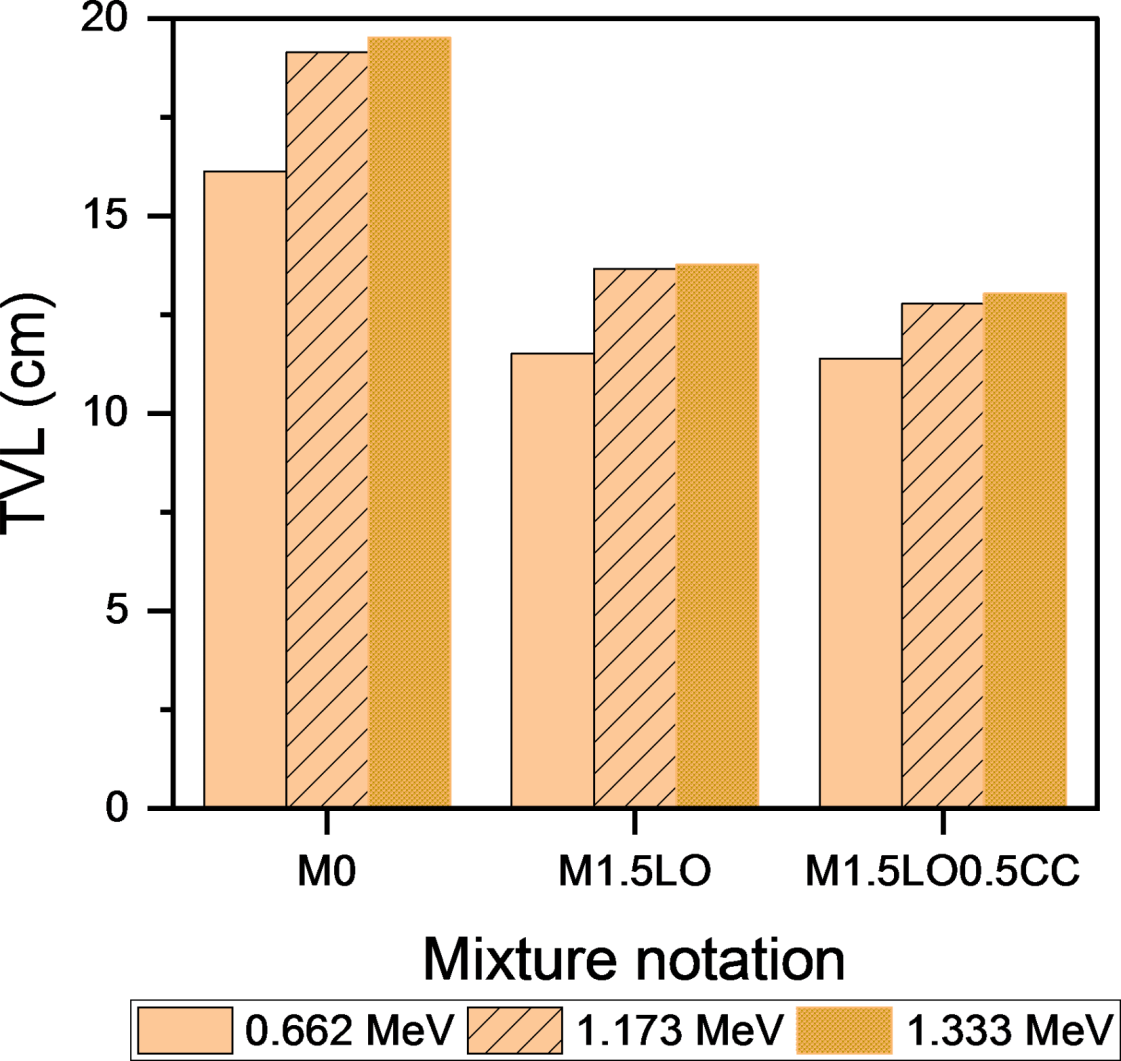
Effect of PbO and PbO/CC on tenth-value layer of cement mortar.

**Fig. 15:**
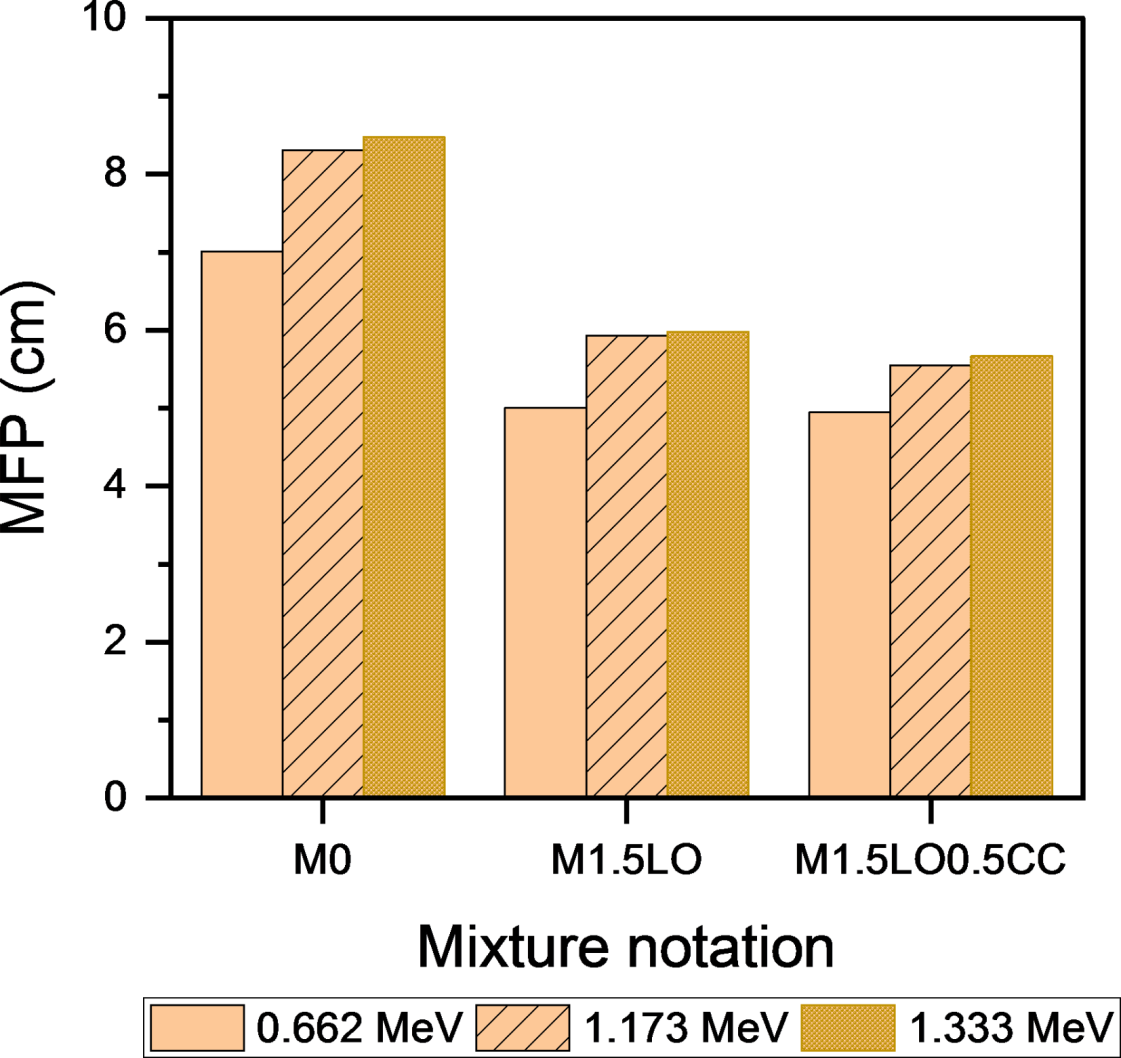
Effect of PbO and PbO/CC on mean-free path of cement mortar.

Figures 13, 14, 15 shows the variations in HVL, TVL, and MFP results as a function of the mixtures M_0_, M_1.5LO_, and M_1.5LO0.5CC_ in the gamma-ray range emitted by Cs and Co isotopes. It is evident from the calculated values that, these radiating parameters are in an inverse relationship with both the linear attenuation coefficient (µ) and the solidified density (ρ). This indicates that, the higher the linear attenuation coefficient, the lower the values of the HVL, TVL and MFP coefficients. For high density lead oxide matrices, the M_1.5LO0.5CC_ mixture was found to exhibit the lowest values at all proposed photon energies. In fact, this tendency is related to this blend can attenuate incident radiation at a shorter distance, since it has a much higher density than its counterparts. According to^6^, to achieve the highest gamma-ray attenuation rate in the range of 662–1333 KeV, it is preferable for mixture to record a low HVL value. Ultimately, cementitious mortar modified by the co-incorporation of PbO and CC displayed superior attenuation for gamma-rays irradiated by Cs and Co isotopes at different photon energies versus the competing samples.

#### Attenuation of fast neutrons

**Fig. 16:**
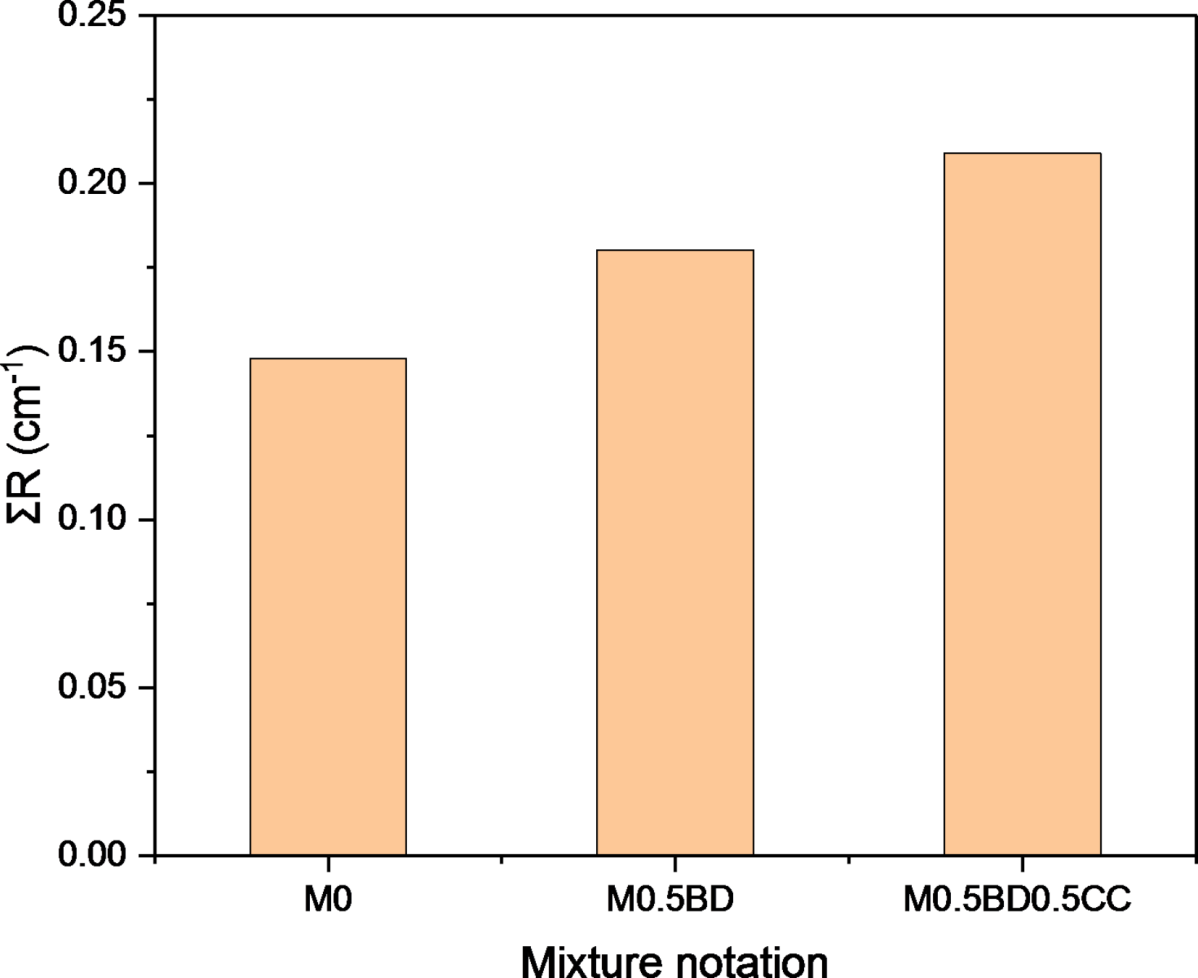
Effect of sole BD and co-incorporation of BD/CC on attenuation of fast neutrons.

Rapid neutrons are difficult to attenuate, as they have a high penetration rate. Dispersion on highly reactive cross-section materials should slow it down. The calculated practical data for Σ_R_ as a function of the studied mixtures, including M_0_, M_0.5BD_ and M_0.5BD0.5CC_ are graphically represented in Fig. 16. The findings show that, the mixes M_0.5BD0.5CC_ and M_0.5BD_, respectively, have superior fast neutron attenuation characteristics than the reference counterpart (M_0_). This is mainly because borax-decahydrate (BD) has a suitable scattering cross-section for moderating fast neutrons.

Similar to Ibrahim et al.^64^, Table 5 summarizes the comparative study between the current findings and those reported in the previous literature. It is evident that the co-combination of lead oxide and borax with calcium chloride has a significant impact on reinforcing of the physico-mechanical characteristics and shielding efficiency of the tested specimens against both gamma rays and fast neutrons emitted from radioactive sources with various photon energies.

**Table 5:** Comparison between the results obtained and their counterparts from previous studies.

LO, %	BD, %	CC, %	Setting time	Compressive strength at 28 d (MPa)	Shielding against gamma	Shielding against neutrons	Ref.
0.05–1	–	–	Acceptable dosage for compressive enhancing is 0.25	Increases up to 0.05	–	–	^21^
–	1–5	–	Increases with dose increasing. As all proportions exceed the specification limits.	Decreases with dose increasing	Enhancing with dose increasing	Enhancing with dose increasing	^65^
–	0–0.5	–	Delayed by increasing the dose	Enhancing with dose increasing alter age	-	–	^66^
5	–	–	Excessively delayed due to overdose by more than 2%	Increased up to 2	Enhancing with dose 5	–	^67^
0.1–0.5	–	–	Increases with dose exceeding 0.2	Increases up to 0.2	Enhancing with dose 0.2	Enhancing with dose 0.2	^56^
1.5	–	0.5	Reduced by 20 min.	Increased by 81%	High	–	Current study
–	0.5	0.5	Reduced by 36 min.	Increased by 5%	–	High	

## Conclusions

In this procedure, the effect of solid inorganic additives on the performance of heavy-density mortar customized for protection against nuclear radiation was evaluated by studying setting times, leachability, bulk density, compressive strength as well as radiation parameters in the field of gamma-rays and fast neutrons utilizing radioactive sources with various photon energies. To achieve the desired target, mixtures were fabricated by the inclusion of different proportions of lead oxide and borax decahydrate. Furthermore, a specific dosage of calcium chloride was co-incorporated with the aforementioned chemicals into a single matrix to accelerate the hydration reaction with enhancing the shielding efficiency. After presenting and discussing the results in detail, we can conclude the following:

1. The w/b ratio of cement pastes containing LO ranged from 0.282 to 0.297; with a decrease of 10.47–5.71% lower than its reference counterpart. Conversely, the inclusion of BD in different proportions almost reduced the water-to-binder ratio from 0.298 to 0.293.
2. The inclusion of borax decahydrate in small doses prolonged the setting times of the pastes compared to their lead oxide-doped counterparts. This is consistent with previous literatures in this regard.
3. The co-incorporation of PbO/CaCl_2_ and Na_2_B_4_O_7_.10H_2_O/CaCl_2_ into the cement matrix shortened the setting time.
4. Upon closer examination, the co-incorporation of PbO with CaCl_2_ in a single mortar ensured a tight closure of the matrix, with the prevention of oxide to leach into the medium.
5. With the inclusion of CaCl_2_ in the preparations impregnated with lead oxide and borax decahydrate, an enhancement in compressive strength has been explored.
6. Compared to their free counterparts, formulations containing 0.5% CaCl_2_ provide higher strength values at all curing periods.
7. Surprisingly, the optimum proportions of Na_2_B_4_O_7_.10H_2_O and PbO incorporated with 0.5% CaCl_2_ into single matrix that provide satisfactory compressive strength at different curing ages are 0.5 and 1.5%, respectively.
8. The findings revealed that, the linear attenuation coefficient increases with the inclusion of PbO in mortar matrix. As previously discussed, the mix M_1.5LO_ exhibited µ value approximately 1.33 folds at photon energy 0.662 higher than the M_0_ mixture.
9. The highest linear attenuation values were achieved with the co-incorporation of PbO with CaCl_2_ in a single matrix, where the blend M_1.5LO0.5CC_ exhibited an increase of 1.41 folds in the field of gamma-ray irradiated by Cesium isotope compared to the reference counterpart.
10. With increasing photon energy from 0.662 to 1.333 MeV, the optimized mixture M_1.5LO0.5CC_ recorded the highest µ values of 1.09 and 1.62 MeV compared to M_1.5LO_ and M_0_ counterparts. Suggesting that, this formulation has the highest hardened density.
11. For fast neutrons attenuation, the formulations M_0.5BD0.5CC_ and M_0.5BD_, respectively, have superior performance than the reference counterpart (M_0_). This is mainly because borax decahydrate has a suitable scattering cross-section to counteract these particles.

## Data Availability

Data is provided within the manuscript. The contact person is Ahmed S. Ouda (ahmed.kamel56@yahoo.com) if someone wants to request the data from this study.
